# Activity in superior parietal cortex during training by observation predicts asymmetric learning levels across hands

**DOI:** 10.1038/srep32133

**Published:** 2016-08-18

**Authors:** Ori Ossmy, Roy Mukamel

**Affiliations:** 1 Sagol School of Neuroscience, Tel-Aviv University, Tel-Aviv 69978, Israel; 2 School of Psychological Sciences, Tel-Aviv University, Tel-Aviv 69978, Israel

## Abstract

A dominant concept in motor cognition associates action observation with motor control. Previous studies have shown that passive action observation can result in significant performance gains in humans. Nevertheless, it is unclear whether the neural mechanism subserving such learning codes abstract aspects of the action (e.g. goal) or low level aspects such as effector identity. Eighteen healthy subjects learned to perform sequences of finger movements by passively observing right or left hand performing the same sequences in egocentric view. Using functional magnetic resonance imaging we show that during passive observation, activity in the superior parietal lobule (SPL) contralateral to the identity of the observed hand (right\left), predicts subsequent performance gains in individual subjects. Behaviorally, left hand observation resulted in positively correlated performance gains of the two hands. Conversely right hand observation yielded negative correlation - individuals with high performance gains in one hand exhibited low gains in the other. Such behavioral asymmetry is reflected by activity in contralateral SPL during short-term training in the absence of overt physical practice and demonstrates the role of observed hand identity in learning. These results shed new light on the coding level in SPL and have implications for optimizing motor skill learning.

It has long been proposed that motion is intrinsically linked to perception such that observing an action activates motor programs that are used to execute the same action^1^. During the last two decades, there has been an increasing interest in identifying the neural underpinning of this perceptual-motor link^2^,^3^,^4^,^5^. Direct recordings in monkeys^6^ and in humans^7^,^8^ demonstrate an overlapping neural representation of observed and executed actions in frontal and parietal regions. Additionally, neuroimaging studies reveal that action observation evokes activity within various regions traditionally associated with motor function such as the primary motor cortex (M1), supplementary motor area (SMA), dorsal premotor cortex (dPMC), supramarginal gyrus, inferior frontal gyrus (IFG) and the superior parietal lobe (SPL)^9^,^10^,^11^. Such evoked activity supports the involvement of these regions in translating perceived actions performed by someone else into a motor plan that can be executed by the observer. More specifically, parieto-frontal regions, that are active during passive action observation, overlap with those engaged during online imitation^12^,^13^,^14^,^15^,^16^. Together, these results suggest a perceptual-motor translation mechanism in the brain, ideally suited for imitation.

Imitation is an important form by which individuals can learn how to perform an action from observing others^17^,^18^,^19^,^20^. Indeed, action observation has been shown to facilitate subsequent executed movements and improve skill performance^21^,^22^,^23^,^24^,^25^,^26^,^27^. The process of learning by observation, was extensively investigated^24^,^28^,^29^,^30^ and supports the idea that the perceptual-motor translation mechanism, which matches observed behaviors with internal motor representations, facilitates subsequent motor performance. Understanding this mechanism can provide a useful account of how we acquire new behaviors, as well as correct our movements^12^,^31^,^32^.

To date, there is no consensus regarding the level of abstraction of what is extracted from observed actions and encoded during the learning process (e.g. kinematics, action goal). Given that the neural representation during physical practice is known to have a contra-lateral bias^33^,^34^, it is not clear whether during action observation the perceptual-motor learning mechanism displays a similar bias. In their associative sequence learning theory, Heyes and Ray argue that action observation engages similar learning processes as overt physical practice, and hence depends on the identity of the observed effector^31^,^35^. This theory was supported by a series of empirical studies in which participants performed a serial reaction time (SRT) task, and performance was facilitated only when subjects used the same effector as the one they observed during passive training^28^,^36^,^37^,^38^. Such evidence are taken to support the notion that the perceptual-motor learning mechanism uses an effector-dependent coordinate frame. However, other recent studies suggest that the perceptual-motor learning mechanism can generalize across effectors. In a behavioral study by Williams and Gribble, subjects passively observed videos of another individual reaching to visual targets in a force field with the right or left hand^39^. Results showed that subsequent right hand performance was facilitated in a similar manner following observation sessions depicting right or left hand. Another study demonstrates shorter reaction times following observation of the same action performed with a different effector^40^. These later results suggest that observed actions evoke neural representations of action goals which are effector independent.

From a neural perspective, neuroimaging studies showed that activation in the parietal lobe is sensitive to observed effectors in a somatotopic manner^2^ and also to visual perspectives (egocentric vs. allocentric)^41^. However little is known as to how the identity of the observed effector modulates neural activity and how such modulation relates to learning by observation. A study by Frey and Gerry showed that activity in the right intraparietal sulcus predicts learning levels following training by observation. However, they used a bimanual sequential task and therefore were not able to outline the role of hand identity (right/left) in the perceptual-motor learning process^42^.

The current study focuses on how learning by observation and its underlying neural mechanisms depend on the identity of the observed effector (right/left hand). To this end we acquired whole-brain functional magnetic resonance imaging (fMRI) data while healthy subjects were engaged in a short-term unimanual learning-by-observation task.

## Results

**Figure 1:**
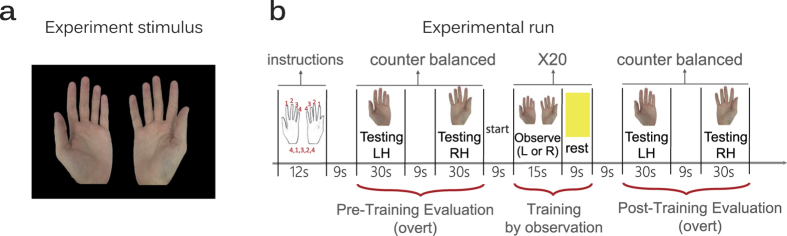
Experimental design. (**a**) Subjects learned a novel sequence of finger movements by observing two virtual hands while one of the hands performed the sequence. (**b**) Each subject completed two experimental runs. In each run, a unique sequence of five digits was presented together with a sketch of the mapped fingers. After instructions, subjects performed the sequence using their right hand (RH) and their left hand (LH) for initial evaluation of performance level. In the training stage subjects passively observed either the right or left virtual hand performing the sequence. After training, subjects repeated the evaluation stage for assessment of changes in performance level.

**Figure 2:**
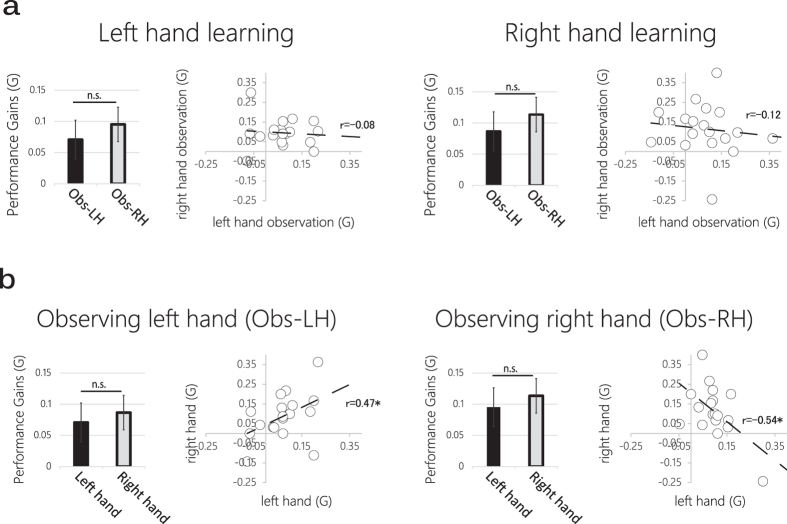
Behavioral results. (**a**) Left hand performance gains were significant relative to baseline following right (Obs-RH; p = 0.002) and left (Obs-LH; p = 0.006) hand observation but not significantly different between the two observation condition. Similarly, right hand performance gains were significant following right and left hand observation but not significantly different between the two observation conditions. (**b**) Following left hand observation there was no significant difference in performance gains between right and left hands at the group level. However, regression analysis on individual subject data revealed a significant positive correlation between left and right hand performance gains. Following right hand observation, there was no significant difference between the two hands at the group level. However, regression analysis on individual subject data revealed a significant negative correlation.

Eighteen healthy subjects were presented with visual input consisting of two virtual hands in egocentric view (Fig. 1a) while either the right or the left virtual hand performed a to-be-learned sequence of finger movements (conditions ‘Obs-RH’ and ‘Obs-LH’ respectively). Performance levels on the sequence of finger movements were evaluated before and after training (see experimental design in Fig. 1b). Left and right hand performance gains (calculated as the accuracy index G; see Methods) were significant in both training conditions – demonstrating significant learning by observation. Within hands, there was no significant difference between performance gains in the Obs-RH and Obs-LH conditions at the group level (p = 0.55 and p = 0.4 for right and left hand respectively). Regression analysis on individual subject data confirmed no significant relationship between performance gains and identity of observed hand. This was true both for left and right hands (See Fig. 2a). We also compared performance gains across hands within each observational training condition. At the group level, there was no significant difference between hands, and this was true both following right or left hand observation (p = 0.68 and p = 0.55 respectively; see Fig. 2b). However, regression analysis on individual subjects revealed a significant correlation between right and left hand performance gains which depended on the identity of observed hand during training. Following left hand observation (‘Obs-LH’), performance gains across hands were positively correlated. Subjects exhibiting high performance gains in the right hand also exhibited high performance gains in the left hand (r = 0.47, p = 0.04; Fig. 2b left panel). Conversely, following right hand observation (‘Obs-RH’), correlation between the two hands was negative (r = −0.54, p = 0.02; Fig. 2b right panel). Thus subjects exhibiting high performance gains in the right hand, exhibited low performance gains in the left hand and vice versa. Taken together, these data demonstrate that although on average at the group level, the identity of observed hand does not affect post-training performance gains, across individual subjects the identity of observed hand has an asymmetric impact on short-term learning across the two hands.

**Figure 3:**
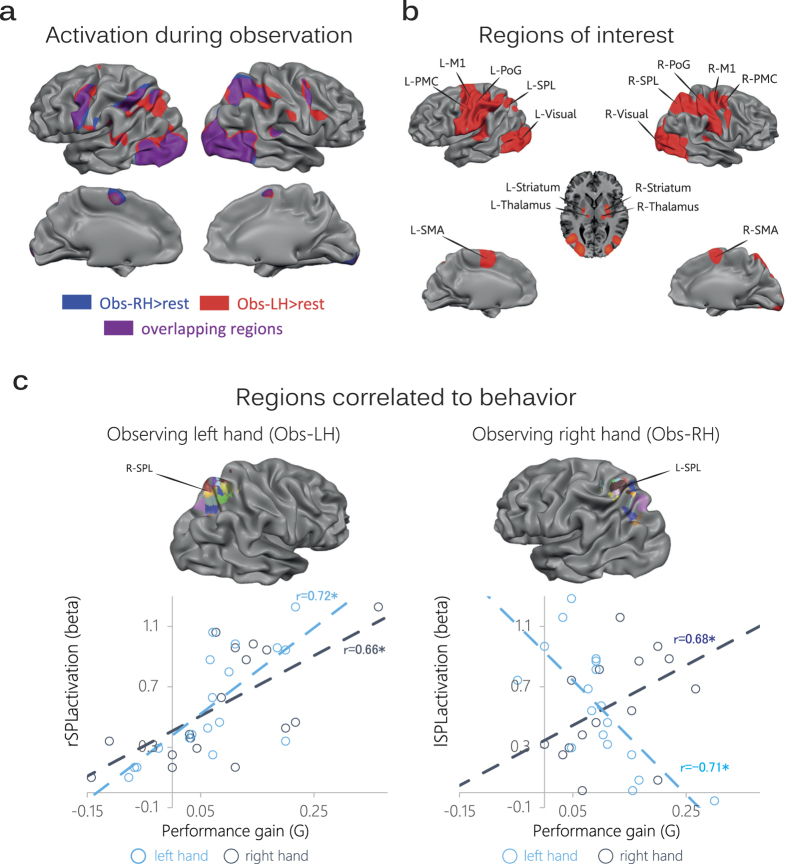
Correlation of neural activity with behavior. (**a**) Random effect multi-subject activation map (N = 18) displaying significant regions obtained from the GLM contrasts: Obs-LH > rest (red) and Obs-RH > rest (blue) in the training stage (q(FDR) < 0.05). Purple regions correspond to overlapping regions between the two contrasts. (**b**) ROI analysis. Random effect multi-subject activation map (N = 18) displaying significant regions obtained from the GLM contrasts right hand execution > rest and left hand execution > rest obtained from the pre-training evaluation period (see Methods). For all analyses, ROIs were defined using this contrast at the individual subject level. (**c**) Across all ROIs, the fMRI signal during training by observation in two regions – right and left SPL - correlated significantly with subsequent performance gains. Each color denotes peak cluster of each subject defined from the localizer (described in panel b). During training by left hand observation, activity in the right SPL correlated both with subsequent left and right hand performance gains. During training by right hand observation, activity in the left SPL showed positive correlation with right hand performance gain and negative correlation with left hand performance gain.

We also explored putative neural networks underlying these behavioral results. First, to examine global differences in activation between left hand observation and right hand observation at the group level, we performed a general linear model (GLM) analysis on the fMRI data obtained during the training stage. The multi-subject map in Fig. 3a demonstrates the high overlap between activation during left and right hand observation relative to baseline (contrast Obs-LH > rest and contrast Obs-RH > rest, red and blue respectively). The direct contrast: Obs-RH vs. Obs-LH, yielded an empty multi-subject map indicating no significant difference in the global activation levels for the two conditions in any particular voxel. These results suggest that at the group level, observing right or left hand finger movements engage spatially overlapping neural networks and at similar activation levels.

Next, we performed a regions of interest (ROI) analysis in individual subjects. In each subject, we defined brain regions engaged during task execution (obtained from the pre-training test period; see multi subject map in Fig. 3b and Methods). For each subject and ROI, fMRI activity during the observational training period was compared against the subsequent behavioral performance gains of each hand (see Methods). We found that across subjects, activity in the right SPL during ‘Obs-LH’ training correlated with left (r = 0.72, p = 7·10^−4^) and right (r = 0.66, p = 2·10^−3^) hand performance gains (See Fig. 3c, left panel; Bonferroni corrected for the 16 regions examined). This is compatible with our observation that across subjects, left and right hand performance gains are positively correlated following training by left hand observation. In the remaining 14 ROIs, no significant correlation values were obtained. We also examined whether fMRI activity level in the same ROIs during ‘Obs-RH’ training period corresponds to subsequent left or right hand performance gains. This time, out of all ROIs only activity in left SPL exhibited a significant positive correlation with right hand performance gain and a significant negative correlation with left hand performance gain (r = 0.68, p = 1.9·10^−3^ and r = −0.71, p = 9.6·10^−4^ respectively; Bonferroni corrected for multiple comparisons; see Fig. 3c, right panel; for Montreal Neurological Institute (MNI) coordinates of ROI centers in individual subjects see Table 1). This is compatible with our behavioral results demonstrating that left and right hand performance gains are negatively correlated following training by right hand observation. These results imply that in the absence of overt physical practice, the SPL in the hemisphere contralateral to the identity of the observed hand plays an important role in the process of short term learning by observation.

## Discussion

Observing and imitating others, has long been recognized as constituting a powerful learning strategy for humans. Previous studies evaluating learning by observation report inconsistent results with respect to the level of abstract representation coded by the perceptual-motor learning mechanism^36^,^37^,^39^,^40^. The present study was designed to determine the effect of identity of the observed effector on learning by observation and trace its underlying neural mechanism.

Behaviorally, the performance gains we found after training support the idea that humans can indeed acquire information about “how” to make movements by mere observation^26^. At the group level, we found that short term motor learning by observation does not depend on the identity of the observed effector suggesting that the perceptual-motor learning mechanism codes actions in an abstract manner. This is consistent with previous studies showing that subjects can acquire a new skill similarly following observed actions performed with different effectors^39^,^40^.

However, closer inspection of individual subject data revealed an unexpected asymmetrical effect of the identity of observed hand. This asymmetry demonstrates that the identity of the observed effector does play a role in short term learning and contributes to the discussion regarding the level of abstract representation during learning by observation. The behavioral asymmetry we found might be a reflection of well documented hemispheric differences in cortical hand representation^43^,^44^,^45^. Such cortical differences are usually associated with hand dominance^33^,^46^ and inter-limb differences in motor control and learning^47^,^48^. Whether or not the asymmetry we report is related to hand dominance is not known and a further study in left-handers is required to clarify this point. Nonetheless, the current results do imply that at least in right-handers, left hand rather than right hand visual input can lead to optimization of short term motor learning by observation.

At the neural level, our results provide strong evidence that in the absence of overt physical practice, the bilateral SPL plays an important role in enhancing short-term learning during observation. The parietal cortex has been previously found active during action observation, demonstrating its engagement in visuo-motor control and imitation learning^6^,^13^,^24^,^49^,^50^. Additionally, it has been suggested that the SPL represents low level aspects (e.g. visual perspective) of the action rather than abstract aspects (e.g. action goal)^12^,^41^,^51^. We extend this view to the context of learning. Although at the group level we did not find significant difference in activation power during left and right hand observation, at the individual subject level activation in SPL depended on the identity of observed hand and correlated with subsequent performance gains. These correlations during observational training on a unimanual task are in agreement with a previous study showing that activity level in parietal regions predicted subsequent performance gains in a bimanual problem solving task^42^. Taken together, the results emphasize the key role of parietal regions in short-term learning by observation of complex action sequences.

Although in the current study we focus on the role of hand identity in learning by observation, the current findings may well have a bearing on the neural underpinning of motor imitation. Previous studies revealed a set of parieto-frontal regions active during both observation and synchronous imitation^13^,^14^,^52^. We found similar regions during the observational training with no significant difference in activation level between observation of right and left hand movements at the group level. A previous imitation study by Aziz-Zadeh *et al.* also demonstrated bilateral activations independent of the identity of the observed hand^52^. Thus, during imitation, the perceptual-motor translation mechanism seems to form a neural representation of the observed action in both hemispheres implying an abstraction with respect to hand identity. However, our finding of hemispheric laterality in SPL’s prediction of subsequent performance gains in individual subjects, suggests that the learning process in SPL is sensitive to the identity of the observed hand.

Finally, the importance of SPL in coding spatial aspects of movements is well established^53^,^54^. In the current study we used a finger movement task that has a strong spatial component. Therefore, additional research will be necessary to assess whether the SPL involvement we report in learning generalizes to other types of tasks with a less dominant spatial factor (e.g. temporal learning tasks). Another key question is to what extent the role of SPL in learning observed actions holds true also for learning motor movements that are not in the repertoire of the observer^55^,^56^,^57^. Future studies should go beyond learning how to combine known movement elements in a new arrangement (e.g. order of finger movements), and examine the acquisition process of entirely new movements (e.g. when children imitate adults^58^).

In summary, by examining not only the group level but also the individual subject level, we show asymmetric performance gains across hands which depend on the identity of the observed hand. Furthermore, we show that the SPL plays a significant role in this asymmetric learning by observation. These findings have implications for the debate regarding the coding level in SPL, and demonstrate that for the purpose of learning in both hands, left rather than right hand observation is preferable.

## Methods

### Subjects

Eighteen healthy subjects (10 females, mean age: 27.4, range: 22–34 years), naïve to the purpose of the study, participated after providing written informed consent. All subjects were right handed according to the Edinburgh Handedness Inventory, with normal or corrected-to-normal vision and no reported cognitive or neurological deficits. Subjects were recruited according to the standard safety criteria for fMRI studies and were compensated by payment (55 NIS/hour). The study protocol was approved by the Ethics Committee of Tel-Aviv University and the Helsinki committee at the Tel-Aviv Sourasky Medical Center, and carried out in accordance with the approved guidelines.

### Procedure and Task

During fMRI scans, subjects completed 5 consecutive experimental runs, in which they learned to perform unique sequences of finger movements. During 3 runs, subjects physically practiced a sequence of finger movements (results reported in a separate manuscript). In two runs, subjects passively learned to perform a sequence of finger movements by observing two virtual hands on the screen while either the left or the right virtual hand performed the sequence (Fig. 1a). Throughout these runs the subjects’ real hands were immobile.

Fingers were numbered from index (1) to little finger (4) and subjects were instructed to learn a unique sequence in each experimental run (one of 5 optional sequences: 4-1-3-2-4, 4-2-3-1-4, 3-2-4-1-3, 3-1-4-2-3, 2-1-4-3-2). The subjects were instructed to learn the sequence and knew that they would be tested on it in pre- and post- evaluation stages. Run order and the finger sequence associated with each run were counter balanced across subjects. Subjects’ finger movements were monitored using motion-sensing MR-compatible gloves (5DT Data Glove Ultra). We programmed a custom-build software, based on the application programming interface provided by 5DT, to extract finger movements from 14 different sensors and control the presentation of the virtual hands (http://www.5dt.com). This allowed us to verify that the subject’s hands are immobile during the training stage and also allowed us to yoke virtual hand movement presented on the screen to real hand movement during the evaluation stages (see below). Small delays in feedback are unavoidable due to sampling rate of the motion-sensing gloves (up to 16 ms) and the refresh rate of the screen (up to 16 ms). We verified that the software itself does not introduce additional delays (time duration for calculating fingers’ position and updating the hands animation was less than 15 ms). Therefore we estimate that delays in visual feedback were no longer than 3 refresh rates in the worst case scenario. During experimental runs, virtual hands were presented with a black background on a screen. Subjects lied supine with their arms to the side of their body and palms facing up. Subjects could not see their real hands during the scans and viewed the screen through a tilted mirror mounted in front of their eyes.

In the beginning of each run (Fig. 1b), subjects were presented with an instructions slide that depicted two hand illustrations with numbered fingers and a 5-digit sequence underneath representing the sequence of finger movements to be learned. The instructions slide was presented for 12 seconds and was followed by a pre-training evaluation stage in which baseline performance level of each hand was separately assessed. During the evaluation, subjects physically performed the required sequence with one hand repeatedly, as fast and as accurate as possible, for 30 seconds (hand order evaluation right\left was counter balanced across all sessions). During the evaluation stage, real-time visual feedback was provided by the virtual hands’ movements which were yoked to the subjects’ real hand movements.

Following the pre-training evaluation stage, a “Start Training” slide appeared for 9 seconds and cued the subjects to the upcoming training stage. In the training stage subjects observed the left or right virtual hand performing the sequence (order of training conditions was counter balanced across subjects). Subject were instructed to observe the screen and refrain from moving their hands. The pace of virtual hand movement during training was constant across both conditions and was set based on the average pace of the subject during previous execution sessions. In cases where the training by observation run was first, the pace was set based on the average pace of previous subjects (range across subject: 4–9 digit sequences per block). Each training block lasted 15 seconds and was followed by a 9-second resting period, in which a blank yellow screen appeared. The training stage consisted of 20 such training blocks (with overall experimental run duration lasting 8 minutes). After the training stage, subject’s performance was evaluated again for 30 seconds in each hand. Similar to the pre-training evaluation, subjects were instructed to repeatedly execute the sequence as fast and as accurately as possible.

### Performance evaluation

In all evaluation stages, we calculated subject’s performance using the formula (1) below:


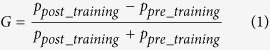


where *p* is the number of times the subject performed a complete sequence with no errors. Therefore, a positive G index reflects improvement in performance. We calculated this performance gain index for each subject, each training condition (Obs-RH, Obs-LH), and each hand (left/right) - allowing us to compare improvement under different motor trainings conditions.

### Behavioral analysis

We used the data from the motion-detection gloves to verify that the subjects did not move their fingers during the observational training conditions. Each sensor of the glove provided the angle of each finger joint (sampling rate = 16 ms) and the subjects always started the training sessions with the hand in the same orientation. The maximal angle of each finger during observational training was not significantly different from the maximal angle during rest. This was true for all fingers of both the right and the left hand (minimal p = 0.42; two-tailed paired t-test; in the main text, unless otherwise stated, all significance tests were two-tailed paired t-test and all correlation tests were Pearson correlation). This analysis rules out an alternative explanation that differences in performance gains (across individuals or conditions) are due to subliminal physical movement of the subjects during observation.

### fMRI data acquisition

Blood oxygenation level dependent (BOLD) images were obtained on a 3 T General Electric scanner with an 8 channel head coil located at the Tel-Aviv Souraski Medical Center, Tel-Aviv, Israel. An echo-planar imaging sequence was used to obtain the functional data (39 ascending interleaved axial slices, 4 mm thickness, slice gaps = 0; TR = 3000 ms; flip angle = 90°; TE = 30 ms; in-plane resolution = 1.72 × 1.72 mm; matrix size = 128 × 128). In addition, anatomical reference was obtained by T1-weighted scan (voxel size = 1 × 1 × 1 mm) for each subject.

### fMRI Preprocessing

All fMRI data were processed using the BrainVoyager QX software (version 2.6, Brain Innovation, Maastricht, Netherlands; http://www.brainvoyager.com). Prior to statistical analysis, a preprocessing procedure was performed on all functional images and included cubic spline slice-time correction, trilinear 3D motion correction, and high-pass filtering (above 0.006 Hz). In addition, we assessed head movements and verified no scans contained head movement exceeding 2 mm in either direction. The 2D functional images were co-registered to the anatomical images and the complete dataset was transformed into the Talairach coordinate system for multi-subject comparisons^59^. Functional data of individual subjects was spatially smoothed (Gaussian filter, FWHM 6 mm) prior to statistical analysis. We also transformed the dataset to Montreal Neurological Institute coordinate system (MNI-305) in order to extract the individual subject peak coordinates that are provided in Table 1.

### ROI Analysis

Regions of interest (ROIs; see Fig. 3b) were defined at an individual subject level. We used general linear model (GLM) contrasts to reveal brain regions active during the pre-training evaluations collapsed across all sessions of each hand: (Pre_Training_right_hand_ > rest) and (Pre_Training_left_hand_ > rest). The resulting maps were corrected by controlling the False Discovery Rate (FDR^60^) and thresholded at q(FDR) < 0.05. For each subject, we defined a sphere, with maximum cluster size of 12 mm radius around the peak activation within each anatomically defined region according to Mai *et al.* 1997. Regions revealed in this analysis include the right and left pre-motor cortex (R-PMc/L-PMc), primary motor cortex (R- 1/L-M1), visual cortex (R-Visual/L-Visual), post-central gyrus (R-PoG/L-PoG), superior parietal lobule (R-SPL/L-SPL), supplementary motor area (R-SMA/L-SMA) and subcortical regions (R-Thlamus/L-Thalamus/R-Striatum/L-Striatum). Next, for each of the eighteen subjects, we calculated separately in each ROI the average fMRI activity level across all significant voxels during training (either Obs-RH or Obs-LH; yielding a vector of 18 values for each ROI and training condition). As the behavioral measure we took the corresponding performance gain (G) in each hand of each subject (See Fig. 3c).

## Additional Information

**How to cite this article**: Ossmy, O. and Mukamel, R. Activity in superior parietal cortex during training by observation predicts asymmetric learning levels across hands. *Sci. Rep.* **6**, 32133; doi: 10.1038/srep32133 (2016).

## Figures and Tables

**Table 1:** MNI locations of each subject’s peak voxels in the left and right SPL ROIs (Fig. 3c).

Subject	Left SPL coordinates	Right SPL coordinates
1	[−31 −63 50]	[24 −65 49]
2	[−22 −74 44]	[25 −76 42]
3	[−28 −64 49]	[17 −65 54]
4	[−18 −65 54]	[25 −57 55]
5	[−25 −65 34]	[25 −65 43]
6	[−32 −51 62]	[24 −53 53]
7	[−20 −52 51]	[23 −56 50]
8	[−34 −51 67]	[13 −63 63]
9	[−25 −66 54]	[25 −66 50]
10	[−24 −59 61]	[21 −54 56]
11	[−29 −58 59]	[30 −57 56]
12	[−34 −64 56]	[22 −63 62]
13	[−27 −52 60]	[22 −60 62]
14	[−31 −51 49]	[31 −53 56]
15	[−31 −54 56]	[29 −54 57]
16	[−29 −63 47]	[24 −65 45]
17	[−35 −72 35]	[28 −66 41]
18	[−23 −55 55]	[24 −54 66]
